# Trismus Nascentium

**Published:** 1879-10

**Authors:** J. T. Banks

**Affiliations:** Prof. Principles and Practice of Medicine, Atlanta Medical College; Atlanta, Ga.


ATLANTA
Medical and Surgical Journal.

VOL. XVII.] OCTOBER—1879. [No. 7.

### Original Communications.

### TRISMUS NASCENTIUM.

By J. T. Banks, M.D., Prof. Principles and Practice of Medicine, Atlanta Medical College.

In reporting this case, I do not propose to add anything to the already doubtful pathology of trismus. The late development of the disease, the probable cause and its successful treatment, are the interesting features that I wish to record.

My little patient was a healthy female, a little above average weight. Parents healthy. The child nursed well, and there was no trouble prior to the attack, except some irregularity in action of the bowels, which was occasionally attended with trismus, especially so if bowels had failed to act often enough. To the latter condition my attention had been called a few days before attack.

Umbilicus healed up entirely and healthy. The history of Tetanus Infantum shows a large majority of cases to come on in the first week and first half of second. It also appears that the chance of recovery is to some extent increased with the age of infant. My patient was three weeks old the day I diagnosed the case trismus neonatorum. The disease, no doubt, was slowly developing for 24 hours before my attention was directed to her condition. At the time I was inquiring after the action of some purgative medicine I had directed for her, and heard that peculiar whine which is a very reliable diagnostic sign of trismus. This whine is so characteristic and constant that no observing physician can hear it without a feeling of awe and dread. In my limited experience with trismus, which is confined to four cases, this peculiar whine or whimper has never failed to attract my attention, and warn me of the fatal *post.*

In seeking for a definite cause for trismus, we are forced to the conclusion that the condition of the nervous centres which give rise to the phenomena in the disease, is not the result of any uniform or definite cause. Nor is there any special definable morbid anatomy of the centres that produce the constant phenomena that trimus presents.

Collus and others charge it to umbilical trouble; Clark and others to imperfect ventilation; West and others to a congested condition of the spinal cord; Nott to occipital pressure on base of brain; some to sudden changes of temperature in certain climates.

Prior to the late war, this disease was quite fatal in parts of the Southern States, especially so among. the negroes, and it was often charged to want of cleanliness, and by some to the careless and filthy condition of the umbilical dressing. Intestinal irritation has been referred to as one of the possible exciting causes. Sum it all up, and we are forced to the conclusion that trismus is the result of a certain condition of the nervous centres, which condition may be the result of any or all exciting causes above referred to. In my little patient, it was no doubt caused by constipation and irritation of the alimentary canal operating as the exciting cause upon a nervous system predisposed to that condition which forms the development of trismus. The occipital condition to which Dr. Nott refers the cause of trismus also existed, but as pressure did not seem to aggravate the spasms and no difference was observed from trial of change of position, I did not regard it as causative. In the treatment of trismus, much difficulty is encountered in getting medicine as well as nourishment in our little patients. Knowing this, the child should be kept well nourished as long as they can swallow, and it is essential to give good attention and use every opportunity for nourishment throughout the attack. To correct the constipation of my patient, I prescribed arom. syr. rhei. and calomel; the syr. rhei. arom. was continued at regular intervals after and into convalescence. To correct the condition of the nervous centres, and control as far as possible the tetanic spasms, I gave brom. potass. and chloral hydrate—to the action of the latter remedy I give much of the credit of cure. I continued to keep up a decided impression for several cays by the bromide and chloral in connection with arom. syr. rhei. every 4 hours. This I continued until all symptoms of spasms ceased and the desire and ability to take nourishment gave proof of convalescence. In pursuing the above treatment, I had the approval and assistance of Drs. Darnell and Caldwell. Cups along the spine were suggested, and being very anxious to save the little patient if possible, I made two attempts to apply them, only to find the disease aggravated by their application. I also rubbed the spine with chloroform (a remedy I have often found valuable in irritations of the spine from reflex nervous action), but I was disappointed in its results. Unmistakable good results followed the use of hydrate chloral in doses from grs. 1 to gr. 5, as condition called for, and repeated as the frequency of the spasms indicated.

